# Supplementary material for: Ni-Catalyzed Enantioselective Intramolecular Mizoroki–Heck Reaction for the Synthesis of Phenanthridinone Derivatives
Source: J Org Chem. 2023 Jun 15;88(13):8203–26. doi: 10.1021/acs.joc.3c00202 (PMC10337041; doi:10.1021/acs.joc.3c00202)
Supplement: Supplementary file 2 — jo3c00202_si_002.zip [file jo3c00202_si_002.zip › Amides(N-H and N-Me)/1a Me-NMe-ArBr/13C Me-NMe-ArBr 1a/1/pdata/1/email_DR-MES5-052 C13 NS 15K_1_1.pdf]

DR-MES5-052 C13 NS 15K
C13APT CDCl3 {D:\nmrusers\malachowski} BM 18

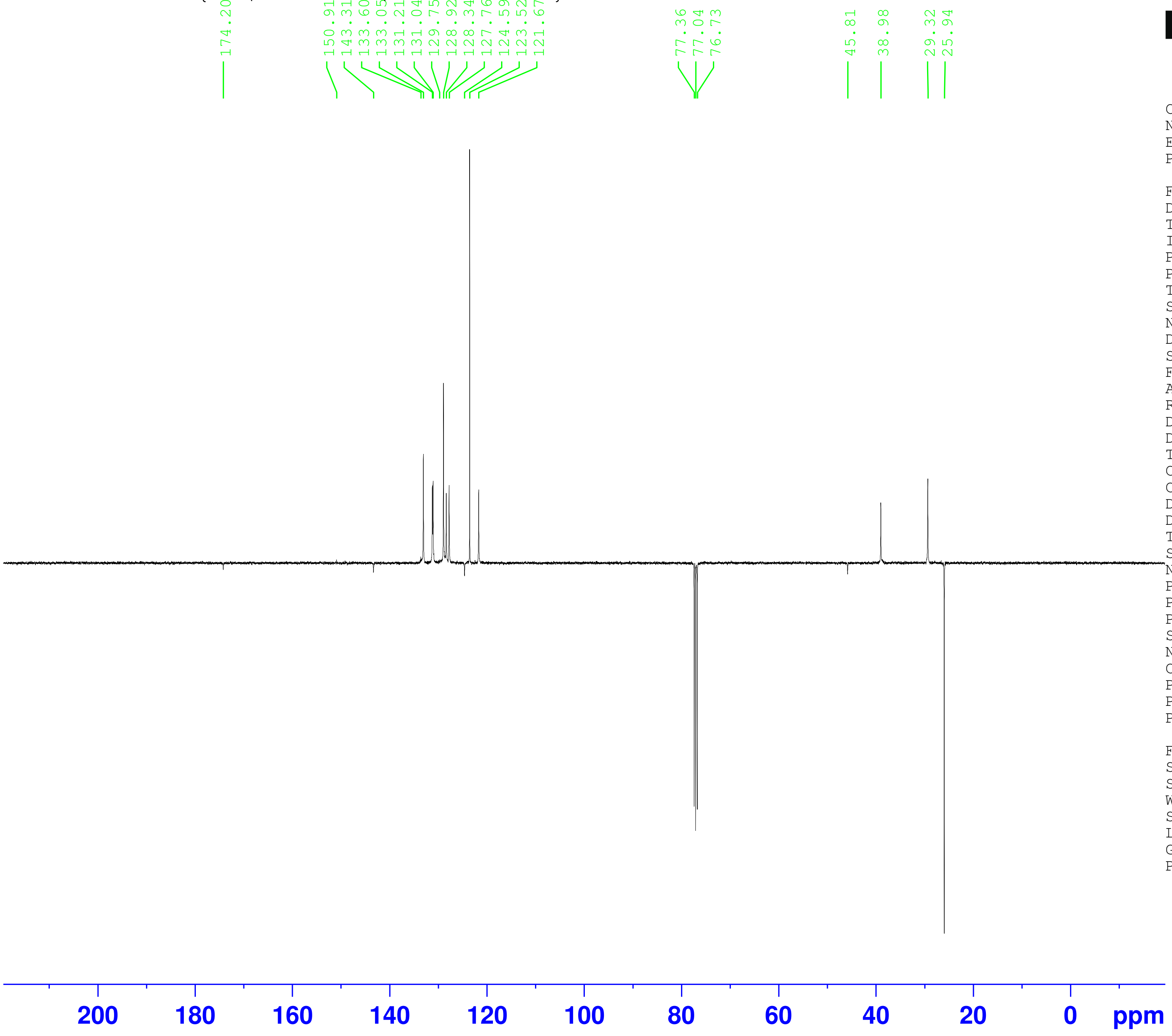

Current Data Parameters

NAME	DR-MES5-052 C13 NS 15K
EXPNO	1
PROCNO	1

F2 - Acquisition Parameters

Date_	20220810
Time	8.22 h
INSTRUM	spect
PROBHD	Z104450_0352 (
PULPROG	jmod
TD	65536
SOLVENT	CDCl3
NS	15000
DS	4
SWH	24038.461 Hz
FIDRES	0.733596 Hz
AQ	1.3631488 sec
RG	203
DW	20.800 usec
DE	6.50 usec
TE	298.0 K
CNST2	145.0000000
CNST11	1.0000000
D1	2.00000000 sec
D20	0.00689655 sec
TD0	1
SFO1	100.6278593 MHz
NUC1	13C
P1	8.00 usec
P2	16.00 usec
PLW1	56.13299942 W
SFO2	400.1516006 MHz
NUC2	1H
CPDPRG[2	waltz65
PCPD2	90.00 usec
PLW2	12.14200020 W
PLW12	0.24368000 W

F2 - Processing parameters

SI	32768
SF	100.6177975 MHz
WDW	EM
SSB	0
LB	1.00 Hz
GB	0
PC	1.40
